# Potent Nrf2-inducing, antioxidant, and anti-inflammatory effects and identification of constituents validate the anti-cancer use of *Uvaria chamae* and *Olax subscorpioidea*

**DOI:** 10.1186/s12906-021-03404-0

**Published:** 2021-09-18

**Authors:** Temidayo D. Popoola, Stephanie T. Guetchueng, Kenneth J. Ritchie, Olufunsho Awodele, Nicola M. Dempster, Oluyemi Akinloye, Satyajit D. Sarker, Amos A. Fatokun

**Affiliations:** 1 Department of Pharmacology, Therapeutics and Toxicology, University of Lagos, Lagos, Nigeria; 2 Centre for Natural Products Discovery (CNPD), School of Pharmacy and Biomolecular Sciences, Liverpool John Moores University, James Parsons Building, Byrom Street, Liverpool, L3 3AF UK; 3 Institute of Medical Research and Medicinal Plants Studies, Ministry of Scientific Research and Innovation, P.O. Box 13033, Yaoundé, Cameroon; 4 Clinical Chemistry Unit, Department of Medical Laboratory Science, University of Lagos, Lagos, Nigeria

**Keywords:** *Uvaria chamae*, *Olax subscorpioidea*, cancer, Antioxidant, Anti-inflammatory

## Abstract

**Background:**

*Uvaria chamae* (UC) and *Olax subscorpioidea* (OS) roots are included in traditional anti-cancer remedies and some studies have identified their chemopreventive/chemotherapeutic potential. This study aimed to identify some cellular/molecular mechanisms underlying such potential and the associated chemical constituents.

**Methods:**

Effect on the viability of cancer cells was assessed using the Alamar Blue assay; ability to modulate oxidative stress was assessed using the 2′,7′-dichlorofluorescein diacetate (DCFDA) assay; potential to modulate Nuclear factor erythroid 2-related factor like-2 (Nrf2) activity was assessed in the AREc32 luciferase reporter cell line; and anti-inflammatory effect was assessed using lipopolysaccharide-induced nitric oxide release model in the RAW264.7 cells (Griess Assay). Chemical constituents were identified through liquid chromatography-mass spectrometry (LC-MS).

**Results:**

Extracts up to 100 μg/ml were non-toxic or mildly toxic to HeLa, AREc32, PC3 and A549 cells (IC_50_ > 200 μg/ml). Each extract reduced basal and peroxide-induced levels of reactive oxygen species (ROS) in HeLa cells. OS and UC activated Nrf2, with UC producing nearly four-fold induction. Both extracts demonstrated anti-inflammatory effects. Chamanetin, isochamanetin, isouvaretin, uvaricin I and other compounds were found in *U. chamae* root extract.

**Conclusion:**

As Nrf-2 induction, antioxidant and anti-inflammatory activities are closely linked with chemoprevention and chemotherapy of cancers, the roles of these plants in traditional anti-cancer remedies are further highlighted, as is their potential as sources of drug leads.

**Supplementary Information:**

The online version contains supplementary material available at 10.1186/s12906-021-03404-0.

## Background

The incidence and prevalence of cancer in the developing world are on the rise [1, 2]. Factors such as demographic changes and growing economies are consequently shifting the disease burden in these regions from infections to non-communicable diseases [2, 3]. Unfortunately, there is an unmet need for effective and affordable approaches to the early detection, diagnosis, prevention and treatment of cancer in these countries [4, 5].

A significant population in these countries, estimated to be up to 80% in some, use herbal remedies as their primary form of health care, even in cancer management [6]. It has been established that natural products contain numerous chemical entities which are pharmacologically active and could act together to affect multiple biological pathways, resulting in the potential efficacy of natural drugs to treat complex pathologies such as cancer [7]. While many plants are employed in traditional medicine as alternative approaches to western medicine in cancer therapy in developing countries [8–11], there is a dearth of robust scientific evidence of the mechanisms of action and safety of many of these remedies.

Preparations from plants have been used as anticancer remedies in regions of Nigeria. *Olax subscorpioideae* Oliv. (Olacaceae) and *Uvaria chamae* P. Beauv. (Annonaceae) are included in decoctions used to treat various cancer subtypes amongst the Ijebu tribe of Nigeria [11]. In that article, *Olax subscorpioidea* was reported to have been cited by 21% of the traditional healers surveyed. Much earlier, Soladoye et al. [9] had reported the widespread use of *U. chamae* and *O. subscorpioidea* as components of several anticancer remedies by traditional healers in South-West Nigeria.

Our previous studies sought to clarify whether or not there are scientific bases for the use of some of these remedies. Our results demonstrated the analgesic and anti-inflammatory activities of these two plants' extracts in animal models [12]. We also showed that these plants possess free radical-scavenging activities, the ability to protect against induced oxidative stress in rodent models, as well as mito-depressive and DNA-damaging properties [13]. These results have so far demonstrated the possible chemopreventive and chemotherapeutic potentials of these plants, which may explain their role in traditional remedies that employ them in the therapy of cancers.

To further understand the mechanistic underpinnings of the effects observed in animal models, the study reported herein employed validated in vitro (cell-based) systems to investigate these plant extracts for their effects on cell viability, oxidative stress and inflammation. We first explored the cytotoxicity, or otherwise, of the extracts, using human cervical adenocarcinoma cells (HeLa), modified human breast cancer cells (AREc32, which is the MCF-7 cell line stably transfected with the antioxidant response element (ARE) reporter plasmid), human prostate cancer cells (PC3) and human lung cancer cells (A549). We then assessed their anti-oxidant activities by examining their ability to reduce or prevent intracellular build-up of reactive oxygen species (ROS) and to induce Nrf-2 activation. Their anti-inflammatory activities were examined in the lipopolysaccharide (LPS)-induced nitric oxide model using RAW264.7 murine macrophages. Chemical profiling of the extracts was then undertaken using Liquid Chromatography-Mass Spectrometry (LC-MS) to reveal their constituents. Our findings provide significant insights into the biological effects of extracts from these plants, the mechanisms underlying the effects, and the compounds that could be responsible for the effects. The work provides some rationale for the inclusion of components of these plants in documented traditional anti-cancer remedies and identifies potential anti-cancer agents.

## Methods

### Plant materials

Fresh roots of *Uvaria chamae* P. Beauv. (Annonaceae) and roots of *Olax subscorpioidea* Oliv. (Olacaceae) were obtained from Mushin market, Mushin, Lagos State, Nigeria. They were identified and authenticated by Mr. T.K. Odewo, a forestry expert in the Department of Botany Herbarium, Faculty of Science, University of Lagos, Akoka, Lagos, Nigeria. Voucher specimens (*U. chamae*, voucher number 3626; *O. subscorpioidea*, voucher number 5577) were deposited in the Herbarium of the Department.

### Extraction

Details of the extraction process have been previously reported [13]. In brief, the freshly harvested plant parts were washed and chopped into smaller pieces and dried in open air for 2 weeks and thereafter ground to powder. Hydroethanolic extracts were prepared by soaking powdered plant parts in 90% ethanol for 72 h and thereafter filtered using Whatman filter paper (~ 9 cm). Each filtrate was poured into drying trays and evaporated to dryness in an oven at 40 °C. The dried extracts were recovered and stored in clean sample bottles [13].

### Chemicals and assay and culture reagents and materials

Chemicals, including MTT, were from Sigma-Aldrich (UK), except where otherwise stated. The Griess Assay reagent (Cat. No. G2930) and the luciferase assay system (Cat. No. E4530) were from Promega (UK), while the DCFDA reagent (Cat. No. ab113851) was from Abcam, UK. Lipopolysaccharide (LPS), S-form, made from *S. typhimurium* (Cat. No. IAX-100-011-M001), was from Caltag Medsystems (UK), while IFN-γ (Cat. No. 485-MI) was from Tocris Bioscience (Biotechne/R&D Systems), UK. Cell culture reagents (basal medium (DMEM), Foetal Calf Serum, L-glutamine, antibiotic-antimycotic (anti-anti) solution (Penicillin/Streptomycin/Amphotericin B), recombinant trypsin solution (TrypLE)) and Alamar blue (Cat. No. DAL1100) were from Thermo Fisher Scientific, while DMEM and G-418 for the AREc32 cells were from Biosera (through Labtech International Ltd., UK). Solvents were from Thermo Fisher, of analytical grade and used without any purification***.*** Specialised black and white 96-well flat-bottom plates were from Greiner Bio-One (UK).

### Cell culture

All cells were grown as adherent monolayer cultures in T75 tissue culture flasks and maintained at 37 °C in a humidified atmosphere of 5% CO_2_ and 95% air. HeLa cells (immortalised human cervical cell line); PC3 cells (human prostate cancer cell line) and A549 (human alveolar basal epithelial adenocarcinoma cell line) were cultured in Dulbecco’s Modified Eagle Medium (DMEM, with 4.5 g/L D-glucose) supplemented with 10% Foetal Calf Serum (FCS), 1% L-Glutamine (2 mM), 1% Non-Essential Amino Acids (NEAA) and 1% anti-anti solution. The MCF-7 AREc32 cell line was cultured in DMEM supplemented with 10% FCS, L-glutamine (2 mM), anti-anti solution and geneticin (G418; 0.8 mg/ml). The RAW 264.7 cell line (murine macrophages) was cultured in DMEM supplemented with 10% FCS and 1% L-glutamine (2 mM) but without antibiotics.

All cells were originally from the European Collection of Authenticated Cell Cultures (ECACC), Salisbury, UK, except AREc32 cells, which were a kind gift to Dr. Kenneth Ritchie from Professor Roland Wolf (University of Dundee, UK), whose lab created the cell line [14].

Cells were detached from flasks through trypsinisation with recombinant trypsin (TrypLE), except the RAW264.7 cells that were detached mechanically through the use of cell scrapers. Cell density was determined through haemocytometer-assisted counting [15].

### Cell viability assays

#### Alamar blue (AB) assay

HeLa, PC3, A549 and AREc32 cells were cultured in their respective media until they reached approximately 80% confluency before using them for the cytotoxicity assay. Cells were seeded into black, microclear 96-well plates at 1 × 10^5^ cells/ml (100 μl/well) and incubated for 24 h at 37 °C and 5% CO_2_ to allow the cells to attach. After 24 h, the medium was discarded and the wells thereafter treated with 100 μl of the different concentrations of extracts prepared in medium. A set of untreated control wells was included in each plate, as well as cells treated with the positive control doxorubicin at up to 20 μM. Following incubation with extracts or doxorubicin for 24 h, 10 μl of Alamar blue solution was added to each well (10% v/v). Following 3 h incubation, fluorescence was quantified at the respective excitation and emission wavelengths of 530 nm and 600 nm using a microplate reader (CLARIOstar Microplate Reader, BMG LABTECH, UK). Each treatment was run in triplicate and each experiment was repeated three times. The percentage cell viability was determined relative to the vehicle-treated control cells [16]:

$$ \%\mathrm{Cell}\ \mathrm{Viability}=\kern1.25em \left[{\mathrm{F}}_1/{\mathrm{F}}_0\right]\times 100 $$

Where F_0_ is the mean fluorescence intensity of the triplicate set of vehicle-treated control wells and F_1_ is the mean fluorescence intensity of each triplicate set of compound- or extract-treated wells.

#### Bright-field imaging

Changes to cell morphology as induced by the various treatments were monitored on an Olympus CKX41 microscope fitted with an Olympus DP71 U-TVIX-2 camera. Images were acquired using the Olympus cellSens entry software.

#### MTT assay

RAW264.7 cells were cultured and seeded in 96-well plates for MTT cytotoxicity assay [16] to evaluate the potential of each plant extract (5–50 μg/ml) and of the anti-inflammatory agents prednisolone (5–50 μM) and diclofenac (5–50 μM) to alter cell viability. Cells were seeded into opaque, microclear, 96-well plates at a density of 2.5 × 10^5^ cells/well (100 μl/well) and left for 24 h in the incubator at 37 °C and 5% CO_2_ to allow the cells to attach. After 24 h, the growth medium in each well was aspirated and the wells were treated with 100 μl of the different concentrations of extracts or standards prepared in growth medium. Following incubation for 24 h, 10 μl of MTT (3-(4,5-dimethylthiazol-2-yl)-2,5-diphenyltetrazolium bromide, 5 mg/ml in PBS) was added to each well. After 3 h incubation at 37 °C, the entire content of each well was discarded and 100 μl of DMSO was added. The absorbance at 570 nm was determined with a microplate reader (CLARIOstar Microplate Reader, BMG Labtech, UK). Each experiment was repeated three times, with three replicates for each treatment in each experiment. The percentage cell viability was determined as percentage of control cells.

%Cell Viability =  [A_1_/A_0_] x 100

Where A_0_ is the mean absorbance of the vehicle-treated (control) wells and A_1_ is the mean absorbance of each triplicate set of extract- or positive control-treated wells.

### Assessment of induction of reactive oxygen species (ROS)

This was done using the DCFDA Cellular ROS Detection Assay Kit. HeLa cells were seeded into a black, clear bottom 96-well microplate at 2.5 × 10^5^ cells/ml (25,000 cells/well at 100 μl/well). The cells were incubated at 37 °C in a humidified atmosphere of 5% CO_2_ and 95% air and allowed to adhere overnight. The medium was thereafter aspirated from each well, followed by rinsing with the buffer provided in the assay kit. The buffer was aspirated and the cells stained by adding 100 μl of a 25 μM DCFDA solution (diluted from a 20 mM stock). Stained cells were incubated for 45 min at 37 °C in the dark. After 45 min, DCFDA solution was removed and the cultures were rinsed with the buffer. The rinse buffer was then aspirated and replaced with 100 μl each of plant extract (5, 25, 50, 100 μg/ml), H_2_O_2_ (10, 50, 200 μM) and ascorbic acid (AA) (50, 25, 50, 100 μM), each in duplicate. Ascorbic acid is an antioxidant and so was used as a positive control. Fluorescence (Ex/Em = 485/535 nm) of the wells was measured (CLARIOstar Microplate Reader, BMG Labtech, UK) at 3 and 24 h following treatment. Background wells (untreated or diluent-treated stained and unstained cells) as well as blank wells (medium only) were included in each experiment. Each experiment was repeated three times. Blank readings were subtracted from all measurements and fold change determined by setting the mean fluorescence value for diluent-treated, stained, control cells to unity and normalising every other mean fluorescence value to it. This assay procedure was to determine whether each of the tested extracts or compounds could modulate, on its own, basal intracellular ROS.

The ability of each extract or ascorbic acid to modulate the increase in intracellular ROS induced by the oxidative stressor hydrogen peroxide was also examined. HeLa cells were grown and treated as described above. Following aspiration of rinse buffer post-DCFDA staining, 50 μl of each plant extract (10, 50, 100, 200 μg/ml) or AA (10, 50, 100, 200 μM) was added to individual wells in duplicate, followed by 50 μl of 100 μM H_2_O_2_, with an appropriate negative control and a peroxide baseline included. The 1:1 (test agent: peroxide) volume additions reduced the final concentration in each well to half of the value indicated earlier in the paragraph. Fluorescence measurements and data analyses were done as described in the preceding paragraph.

### Nrf2/ARE activation assay

The ability of the extracts to induce Nuclear factor erythroid 2 (NF-E2)-related factor like-2 (Nrf2) activity was determined in a luciferase reporter assay by using the AREc32 cells (stable human mammary MCF-7-derived reporter cell line with a luciferase reporter gene construct that is under the control of the rat Glutathione-S-Transferase (*Gsta2*) Antioxidant Response Element (ARE) promoter, with eight copies of the ARE in the promoter region [14]). The transcriptional regulatory element ARE is involved in the activation of genes that code for a number of antioxidant proteins and enzymes that are protective against oxidative insults. The induction of the genes is controlled by the transcription factor Nrf-2, whose activity is usually repressed by the inhibitory factor Kelch-like ECH associated protein 1 (Keap1) that facilitates its degradation, while electrophilic agents prevent Keap1 from targeting Nrf2 for degradation [14]. The AREc32 cell line is, therefore, used to examine whether an anti-cancer drug or drug candidate can induce ARE-driven gene expression, as induction of ARE causes luciferase activity.

AREc32 cells were seeded into 96-well plates at a density of 1.2 × 10^4^ cells per well. After 24 h, the medium was discarded and 100 μl of medium containing each plant extract was added at a range of concentrations into receiving wells. Then, after another 24 h of incubation, medium was discarded, cells were washed with phosphate-buffered saline (PBS) and 20 μl of luciferase lysis buffer was added to each well, followed by a freeze-thaw cycle to achieve complete lysis. The cell lysate was then aspirated and dispensed into a white 96-well plate. 100 μl of a luciferase reporter substrate was then added to each well and luminescence measured (CLARIOstar Microplate Reader, BMG Labtech, UK) immediately. The level of luciferase activity for each treatment was compared to the basal level of luciferase activity in control cells and presented as a fold increase. *Tert*-butylhydroquinone (tBHQ; 25 μM) served as positive control. Each experiment was repeated three times, with three replicates in each experiment.

### Inhibition of nitric oxide (NO) production (Griess assay)

Following MTT assay and confirmation of the non-toxicity of the tested concentrations, RAW 264.7 cells were seeded into 96-well opaque plates at a density of 2.5 × 10^5^ cells/well; after 24 h of incubation, the culture medium was replaced with 90 μl of medium containing different concentrations of extracts (for final concentrations of 5, 10, 25, 50 μg/ml), diclofenac or prednisolone (for final concentrations of 5, 10, 25, 50 μM). Following 1 h of incubation, 10 μl of 10 μg/ml LPS was added to the wells containing the concentrations of the extracts or positive control compounds (prednisolone and diclofenac), as well as to the vehicle-treated wells (negative controls without LPS or extract/compound were also included). The final concentration of LPS to which cells were exposed was 1 μg/ml. The cells were cultured for a further 24 h, after which nitric oxide (NO) levels were assessed by nitrite quantification as previously described [17] using Promega’s Griess Reagent System. Briefly, experimental samples (50 μl supernatant from the wells) as well as nitrite concentration standards prepared from the supplied nitrite stock solution were incubated in the dark, at room temperature, with 50 μl of suphanilamide (1% in 5% H_3_PO_4_). After 10 min of incubation, 50 μl of napthylethylenediamine (0.1% in distilled H_2_O) was added and a further 10 min incubation was carried out in the dark at room temperature. Absorbance was read at 550 nm on a microplate reader (CLARIOstar Microplate Reader, BMG Labtech, UK). Each experiment was repeated three times, with each treatment assessed in triplicate in each experiment. The nitrite concentration was determined by comparison to a nitrite standard reference curve.

The ability of the extracts to inhibit NO production following LPS stimulation potentiated by Interferon gamma (IFNγ) was also investigated. RAW 264.7 cells were seeded into 96-well opaque plates at a density of 2.5 × 10^5^ cells/well; after 24 h of incubation, the culture medium was replaced with 80 μl medium containing different concentrations of extracts (final concentrations of 5, 10, 25, 50 μg/ml), diclofenac or prednisolone (final concentrations of 5, 10, 25, 50 μM). Diclofenac and prednisolone are anti-inflammatory agents and so were used as positive controls. Following 1 h of incubation, 10 μl of 1 μg/ml LPS was added to the wells, followed by 10 μl of 50 ng/ml IFNγ. The final concentrations of LPS and IFNγ were 0.1 μg/ml and 5 ng/ml, respectively, and negative controls were included. Cells were cultured for a further 24 h, after which nitric oxide (NO) levels were assessed by nitrite quantification as described earlier.

### Liquid chromatography-mass spectrometry (LC-MS) analyses

Analytical high-pressure liquid chromatography (HPLC) experiments were performed on a Thermo Scientific UPLC Dionex Ultimate 3000 series (Thermo Scientific, UK) equipped with a binary pump, an autosampler, a column chamber, a degasser and a UV/DAD detector. Extracts prepared in methanol were analysed using a Thermo Scientific Hypersil GOLD (150 × 4.6 mm, 5 μm; Runcorn, UK) C_18_ column. The flow rate was set at 1 ml/min; solvent gradients of 50–100% MeOH over 30 min and 15–90% acetonitrile (ACN) over 30 min were used to ensure optimal peak separations for UC and OS, respectively. The column temperature was set at 25 °C. The chromatogram for each extract was acquired at 280 nm.

The LC analysis was performed using a Waters 2695 Separation Module (Alliance) system (Milford, MA, USA) coupled to a Waters 2487 Dual Absorbance Detector. Separation of compounds was performed on a Thermo Scientific Hypersil GOLD (150 × 4.6 mm, 5 μm; Runcorn, UK) C_18_ column using a linear gradient of 50–100% MeOH over 30 min and 15–90% ACN over 30 min, for UC and OS, respectively. Injection volume was 25 μl; flow rate was 1 ml/min. Mass spectra were acquired using a Waters Micromass LCT Premier Mass spectrometer (Milford, MA, USA) with an electrospray interface in the positive mode (ESI+), and an ion spray of 3 kV was used; sample cone voltage was set at 30 V; mass (m/z) scan was set within the range of 100–1500. Data acquisition and analysis were performed with MassLynx software (Waters, Milford, MA, USA).

### Statistical analyses

All data are expressed as mean ± SEM. In order to assess the statistical significance of the differences between means, one-way analysis of variance (ANOVA) was conducted, followed by Dunnett/Tukey multiple comparisons post-hoc test, as applicable. GraphPad Prism® 6 (GraphPad Software Inc., CA, USA) was used to accomplish statistical tests. Values of *p* < 0.05 were considered statistically significant.

## Results

### Effects of extracts on cell viability

**Fig. 1:**
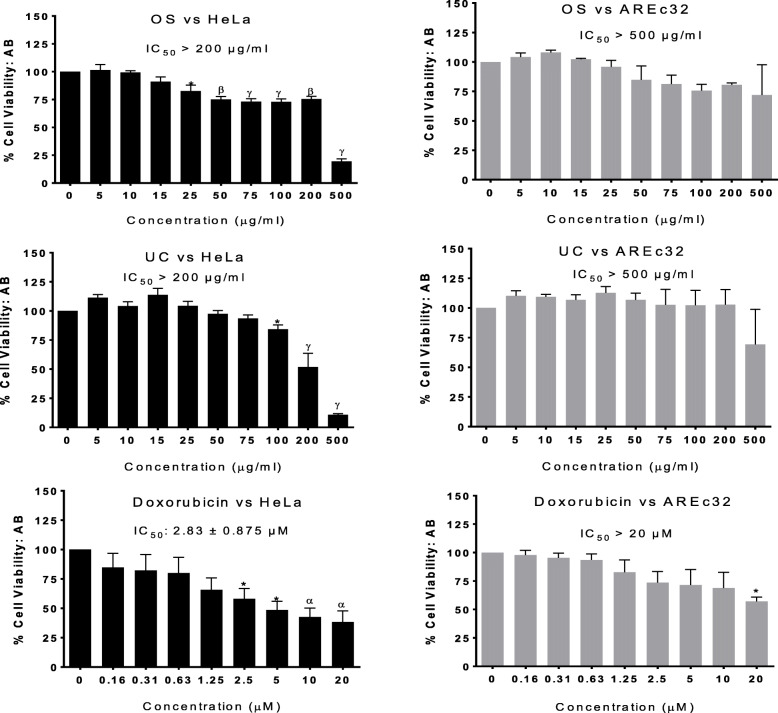
Cell viability (%) (Alamar blue assay) in HeLa cells and AREc32 (MCF-7) cells following 24 h exposure to *O. subscorpioidea*, *U. chamae* and doxorubicin. Each bar represents mean ± SEM (*n* = 3); *p < 0.05, ^α^*p* < 0.01, ^β^*p* < 0.001, ^γ^*p* < 0.0001 vs. control using one-way ANOVA followed by Dunnett’s post hoc multiple-comparisons test

It was important to identify for the extracts both their non-toxic range and toxic range of concentrations in order to be able to appropriately design subsequent assays and interpret their data. The effect of a substance on cell viability depends on the type of cell used, so we explored a range of human cancer cell lines grown as monolayer cultures: HeLa (cervical adenocarcinoma cell line); AREc32 (a variant of the human mammary tumour cell line MCF-7); A549 (alveolar basal epithelium adenocarcinoma cell line); and PC3 (prostate cancer cell line). The cultures were treated with the concentrations of each extract for 24 h, consistent with the duration of the assays that the cells were to be subsequently used for. Following treatments, the Alamar Blue reagent was used to quantify changes to viability, complemented with microscopic monitoring and capture of any associated morphological changes. The anti-cancer drug doxorubicin was included as a positive control. The effects of the extracts and doxorubicin on the viability of HeLa and AREc32 cells are shown in Fig. 1, while their effects on PC3 and A549 cells are shown in Table 1. We first demonstrated that the viability of each of the cell types could be impaired through exposure to a known toxic agent (doxorubicin), as doxorubicin reduced their viability in a concentration-dependent manner, and the effects at higher doxorubicin concentrations were significant.

**Table 1:** Percentage viability values for A549 and PC3 cells obtained in Alamar blue assay following 24 h exposure to *O. subscorpioidea, U. chamae* and doxorubicin

Test Conc. (μg/ml)	***O. subscorpioidea***		***U. chamae***		Doxorubicin		
					Conc. (μM)	A549	PC3
	A549	PC3	A549	PC3			
5	97.10 ± 1.24	97.46 ± 3.75	97.29 ± 1.50	92.55 ± 4.72	0.16	79.09 ± 11.39	91.90 ± 5.69
10	91.93 ± 1.67	93.77 ± 3.41	102.75 ± 3.33	93.25 ± 4.16	0.31	70.57 ± 9.02	83.34 ± 9.64
15	76.15 ± 1.47*	93.32 ± 1.85	104.67 ± 6.32	92.76 ± 4.77	0.63	66.76 ± 8.39*	83.97 ± 12.27
25	66.95 ± 2.25*	87.40 ± 4.62	102.61 ± 5.24	92.84 ± 7.03	1.25	62.98 ± 7.42*	76.50 ± 8.14
50	64.25 ± 3.43*	84.29 ± 5.62	105.88 ± 1.90	88.00 ± 5.15	2.5	60.04 ± 7.23*	68.52 ± 2.02*
75	61.17 ± 3.89*	81.30 ± 6.63	95.21 ± 6.59	86.93 ± 4.72	5	58.87 ± 8.34^α^	58.08 ± 0.20^α^
100	59.17 ± 4.40*	82.51 ± 6.08	92.77 ± 10.21	83.05 ± 4.31	10	56.25 ± 8.43^α^	57.23 ± 2.03^β^
200	61.30 ± 3.81*	82.87 ± 9.09	53.08 ± 7.58^γ^	56.91 ± 5.08^γ^	20	52.08 ± 7.47^α^	54.01 ± 1.15^β^
500	10.13 ± 0.62^γ^	20.36 ± 5.27^γ^	22.50 ± 10.02^γ^	21.04 ± 4.16^γ^			
**IC**_**50**_	**≈ 270**	**≈ 304**	**≈ 274**	**≈ 273**		**> 20**	**> 20**

Cell viability (%) of A549 and PC3 cells following 24 h exposure of the cells to varying concentrations of test agents as measured by the Alamar blue assay. Values are presented as mean ± SEM (*n* = 3). *p < 0.05, ^α^*p* < 0.01, ^β^*p* < 0.001, ^γ^*p* < 0.0001 vs. control (100% viability) using one-way ANOVA followed by Dunnett’s post hoc multiple-comparisons test.

**Fig. 2:**
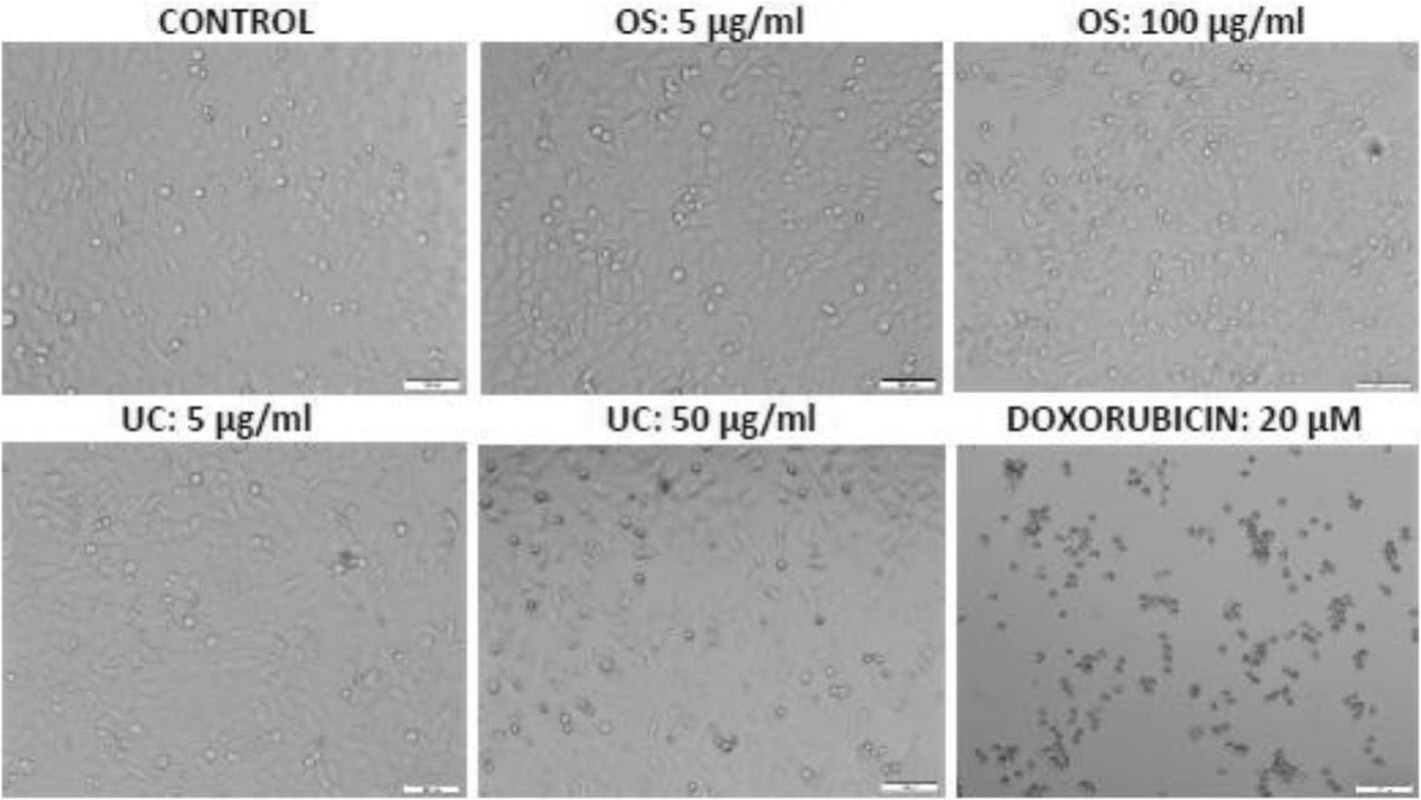
Brightfield photomicrographs showing the non-toxic effects of OS and UC (up to 100 μg/ml) and the toxic effect of the positive control doxorubicin on the morphology of HeLa cells. Cultures were exposed to each agent for 24 h. Scale bar = 100 μm

In HeLa cells (Fig. 1), higher concentrations of OS and UC reduced cell viability (most of them mildly), but the IC_50_ values were greater than 200 μg/ml. The extracts did not affect the viability of the AREc32 (MCF-7) cells (IC_50_ > 500 μg/ml). In the A549 cells, even though some toxicity of the extracts began to be observed at relatively low concentrations, the IC_50_ for each of the extracts was greater than 250 μg/ml (Table 1). In the PC3 cells, significant toxicity only occurred at 500 μg/ml for OS (IC_50_ > 300 μg/ml) and from 200 μg/ml for UC (IC_50_ > 250 μg/ml) (Table 1). The morphological changes observed following the treatments were consistent with the viability changes. Photomicrographs of control cultures and cultures of non-toxic or mildly toxic treatments revealed confluent cells in a healthy organisation, while cultures of overtly toxic treatments, exemplified by the highest tested concentration (20 μM) of the standard, doxorubicin (which elicited concentration-dependent toxicity), revealed loss of confluency and thus of cell-cell contacts, with some or most of the remaining cells appearing rounded and shrunken (Fig. 2).

Using HeLa cells, we investigated whether a longer treatment duration (48 h) could induce more significant cytotoxicity. However, there were no differences between the viability values obtained following 24 h treatments with each extract and those obtained following 48 h treatments (Figure S1 – supplementary data).

### Extracts reduced basal and hydrogen peroxide-induced intracellular levels of ROS

**Fig. 3:**
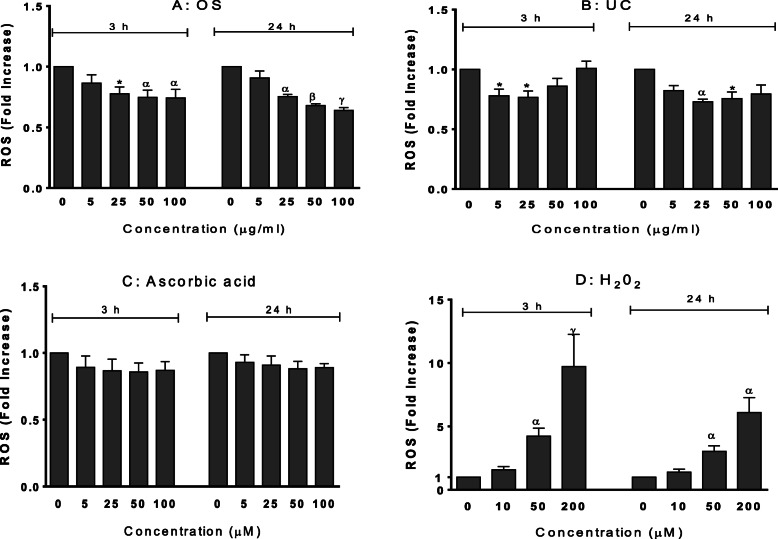
Intracellular Reactive Oxygen Species (ROS) levels presented as fold increases in fluorescence (Relative Fluorescence Unit, RFU) relative to the negative control, following 3 h and 24 h exposures of HeLa cells to extracts of *O. subscorpioidea* (**A**), *U. chamae* (**B**), and to Ascorbic acid (**C**) and Hydrogen peroxide (**D**). Each bar represents Mean ± SEM (*n* = 3); ^*^p < 0.05, ^α^*p* < 0.01, ^β^*p* < 0.001, ^γ^*p* < 0.0001 versus negative control (nil concentration) using one-way ANOVA followed by Dunnett’s post hoc multiple-comparisons test

OS and UC significantly reduced, at some or all of the concentrations, basal intracellular ROS levels in HeLa cells at 3 and 24 h, whereas ascorbic acid did not affect the basal ROS levels (Fig. 3). H_2_O_2_ caused concentration-dependent elevations of intracellular ROS levels, which were significant at 50 μM and 200 μM, with the latter producing nearly a 10-fold increase compared to the negative control (Fig. 3).

**Fig. 4:**
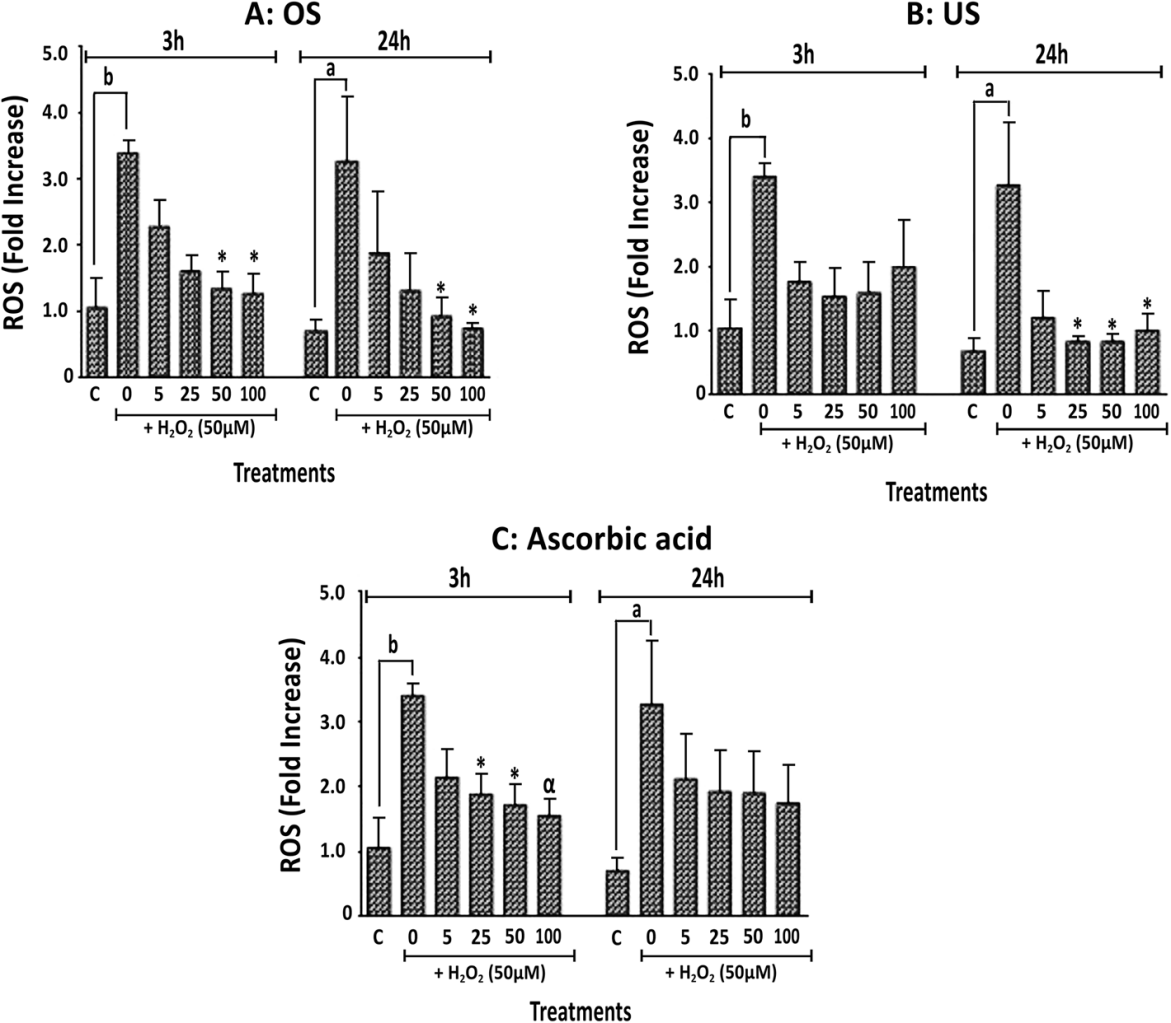
Intracellular Reactive Oxygen Species (ROS) levels as fold increases in fluorescence (Relative Fluorescence Unit, RFU) relative to the negative control (indicated as “C”), following 3 h and 24 h exposures of HeLa cells to extracts of *O. subscorpioidea* (**A**), *U. chamae* (**B**), and to Ascorbic acid (**C**), each in the presence of Hydrogen peroxide (50 μM). Each bar represents Mean ± SEM (n = 3); ^a^*p* < 0.05, ^b^*p* < 0.01 versus vehicle (negative) control (indicated as “C”); *p < 0.05, ^α^*p* < 0.01 versus peroxide only-treated control using one-way ANOVA followed by Dunnett’s post hoc multiple-comparisons test

The ability of each extract to inhibit peroxide-induced elevation of intracellular ROS was then investigated. OS, UC and ascorbic acid each attenuated the elevation of intracellular ROS induced by hydrogen peroxide (10 μM) (Fig. 4). The effects of OS on the 3.5-fold increase in ROS induced by peroxide were clearly concentration-dependent, and significant at 50 μg/ml and 100 μg/ml for both time points, and at the 24 h time point its 100 μg/ml completely inhibited the effect of peroxide. The effects of UC were only significant from 25 μM at the 24 h time point. Ascorbic acid produced significant effects only at the 3 h time point.

### OS and UC induce Nrf2/ARE activation

**Fig. 5:**
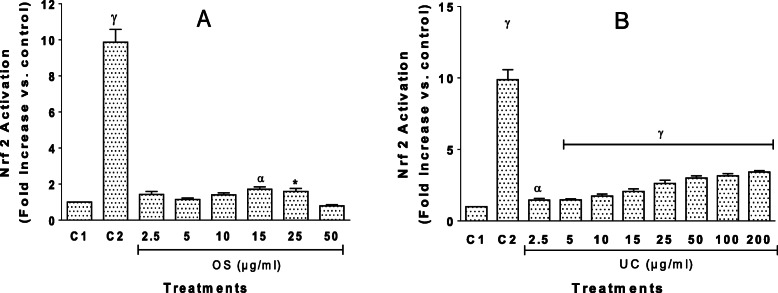
Nrf2 activation following exposure of AREc32 luciferase reporter cells to extracts of *O. subscorpioidea* (**A**) and *U. chamae* (**B**). Each bar represents Mean ± SEM (*n* = 3); C1 denotes negative (unstimulated) control, while C2 denotes positive control, which is the antioxidant Nrf2 activator *tert*-butylhydroquinone (tBHQ) at 25 μM; *p < 0.05, ^α^*p* < 0.01, ^γ^*p* < 0.0001 versus negative control (C1) using one-way ANOVA followed by Dunnett’s post hoc multiple-comparisons test

The Nrf2-ARE signalling pathway is a key endogenous cellular defence system against oxidative stress and some antioxidants are known to act by activating this system [18]. We, therefore, tested the ability of each extract to activate the Nrf-ARE pathway. This was done using the stable AREc32 luciferase reporter cell line, derived from the human mammary MCF-7 cell line, with its luciferase reporter gene construct under the control of the rat Glutathione-*S*-Transferase (*Gsta2*) Antioxidant Response Element (ARE) promoter [14]. The extract concentrations used had been determined to be non-toxic to the AREc32 cells, evidenced by their not reducing viability for any more than 10% in the Alamar Blue viability assay. As shown in Fig. 5, the antioxidant Nrf2 activator *tert*-butylhydroquinone (tBHQ) (25 μM), used as a positive control, potently induced a significant, almost ten-fold, increase in Nrf2 activity compared to the negative (unstimulated) control. OS significantly activated Nrf2 at 15 and 25 μg/ml (Fig. 5A). Remarkably, UC produced a significant and concentration-dependent activation of Nrf2, an effect that was significant even at a concentration as low as 2.5 μg/ml (*P* < 0.01), with the highest concentration tested (200 μg/ml) producing a more than 3-fold activation (*P* < 0.0001) of Nrf2 activity (Fig. 5B).

### Extracts of OS and UC inhibited lipopolysaccharide (LPS)-induced nitric oxide (NO) production

**Fig. 6:**
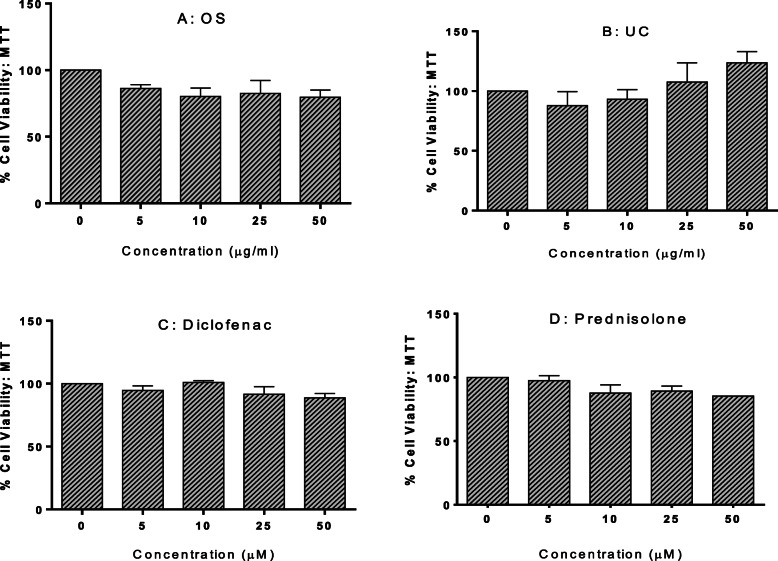
Cell viability (%) of RAW264.7 murine macrophage cells following 24 h exposure to extracts of *O. subscorpioidea* (**A**), *U. chamae* (**B**) and to Diclofenac, (**C**) and Prednisolone (**D**), as quantified using the MTT assay. Each bar represents mean ± SEM (*n* = 3); *p < 0.05, ^α^*p* < 0.01 vs. control using one-way ANOVA followed by Dunnett’s post hoc multiple-comparisons test

Figure 6 shows the results of the MTT assay evaluating the potential effects of the extracts, diclofenac and prednisolone on RAW264.7 cell viability. The results demonstrate the non-toxic effects of the extracts and chemicals on the cells at the test concentrations.

**Fig. 7:**
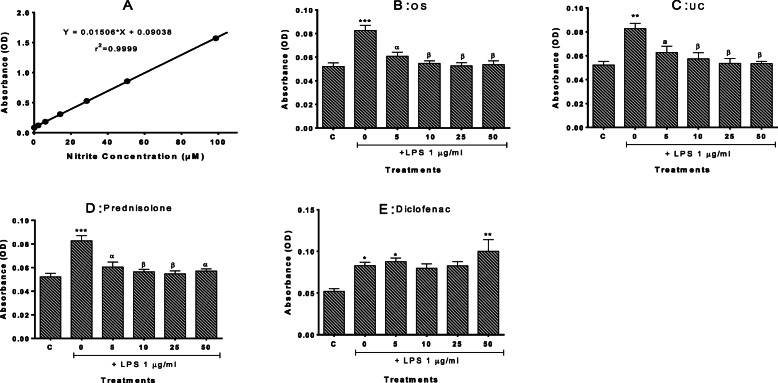
Nitric oxide (NO) release following 24 h stimulation of RAW264.7 murine macrophage cells with lipopolysaccharide (LPS) in the absence or presence of extracts of *O. subscorpioidea* (**B**), *U. chamae* (**C**), and of Prednisolone (**D**) and Diclofenac (**E**). **A** describes the standard nitrite curve obtained. Each bar represents Mean ± SEM (*n* = 3); ^*^p < 0.05, **p < 0.01, ***p < 0.001 versus vehicle-treated control (C); ^a^*p* < 0.05, ^α^*p* < 0.01, ^β^*p* < 0.001 versus LPS-treated cultures using one-way ANOVA followed by Dunnett’s post hoc multiple-comparisons test

Figure 7 summarises the results of the subsequent assay evaluating the inhibition of NO production in RAW264.7 cells by the study plants at the concentrations confirmed to be non-toxic to the cells. LPS (1 μg/ml) significantly increased the levels of the pro-inflammatory mediator NO compared to the negative control (*P* < 0.001). OS and UC were significant inhibitors of the LPS-induced NO. The effects of these extracts were similar to that observed with the positive control prednisolone, where NO levels were restored to control levels following treatment with the higher concentrations of the agents.

**Fig. 8:**
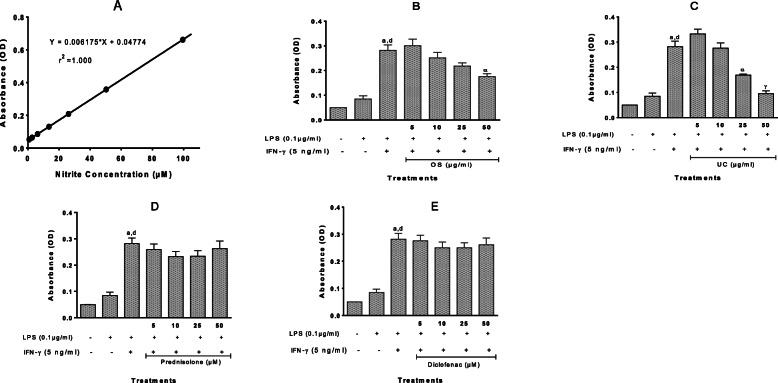
Nitric oxide (NO) release following 24 h stimulation of RAW264.7 murine macrophage cells with LPS+ IFN-γ in the absence and presence of extracts of *O. subscorpioidea* (**B**), *U. chamae* (**C**), and of Prednisolone (**D**) and Diclofenac (**E**). **A** describes the standard nitrite curve obtained. Each bar represents Mean ± SEM (*n* = 3); ^a^*p* < 0.001 versus vehicle-treated control; ^d^*p* < 0.001 versus LPS-treated control; *p < 0.05, ^α^*p* < 0.01, ^γ^*p* < 0.001 versus LPS+ IFN-γ-treated cultures using one-way ANOVA followed by Dunnett’s post hoc multiple-comparisons test

However, the linear correlation between absorbance values and nitrite levels as shown in Fig. 7A (r^2^ = 0.9999) suggests that the levels of nitric oxide (indicated by nitrite levels) induced by LPS alone (absorbance of about 0.08, correlated to very low nitrite levels) were quite low. We, therefore, potentiated LPS-induced NO release with the addition of IFN-γ at 5 μg/ml [19] (the concentration of LPS was reduced 10-fold to 0.1 μg/ml) (Fig. 8). Following this potentiation, NO levels induced by IFN-γ-potentiated LPS were at least 3-fold higher than those induced by LPS alone, with the absorbance values for the potentiated effect correlating with average nitrite concentrations of nearly 40 μM. Each extract produced significant, concentration-dependent inhibition of NO release following potentiated LPS stimulation, which was significant at 50 μg/ml for OS and 25 and 50 μg/ml for UC, although neither of them restored NO levels to that of the control. The standards prednisolone and diclofenac showed no significant effects (Fig. 8).

### Chromatographic and mass spectrometric analyses

**Fig. 9:**
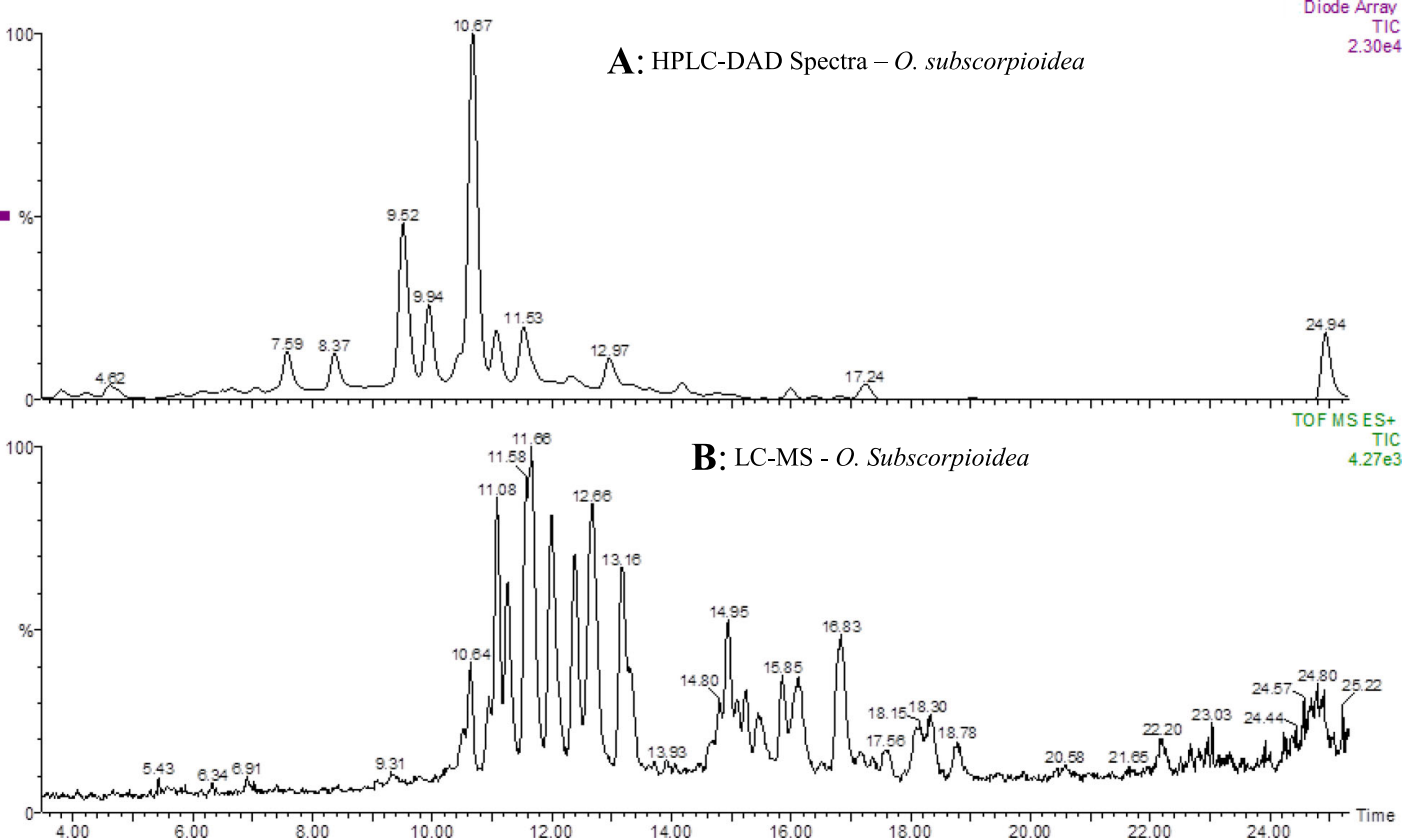
Chromatographic data for *O. subscorpioidea* root extract. **A** HPLC-DAD, **B** LC-MS

**Fig. 10:**
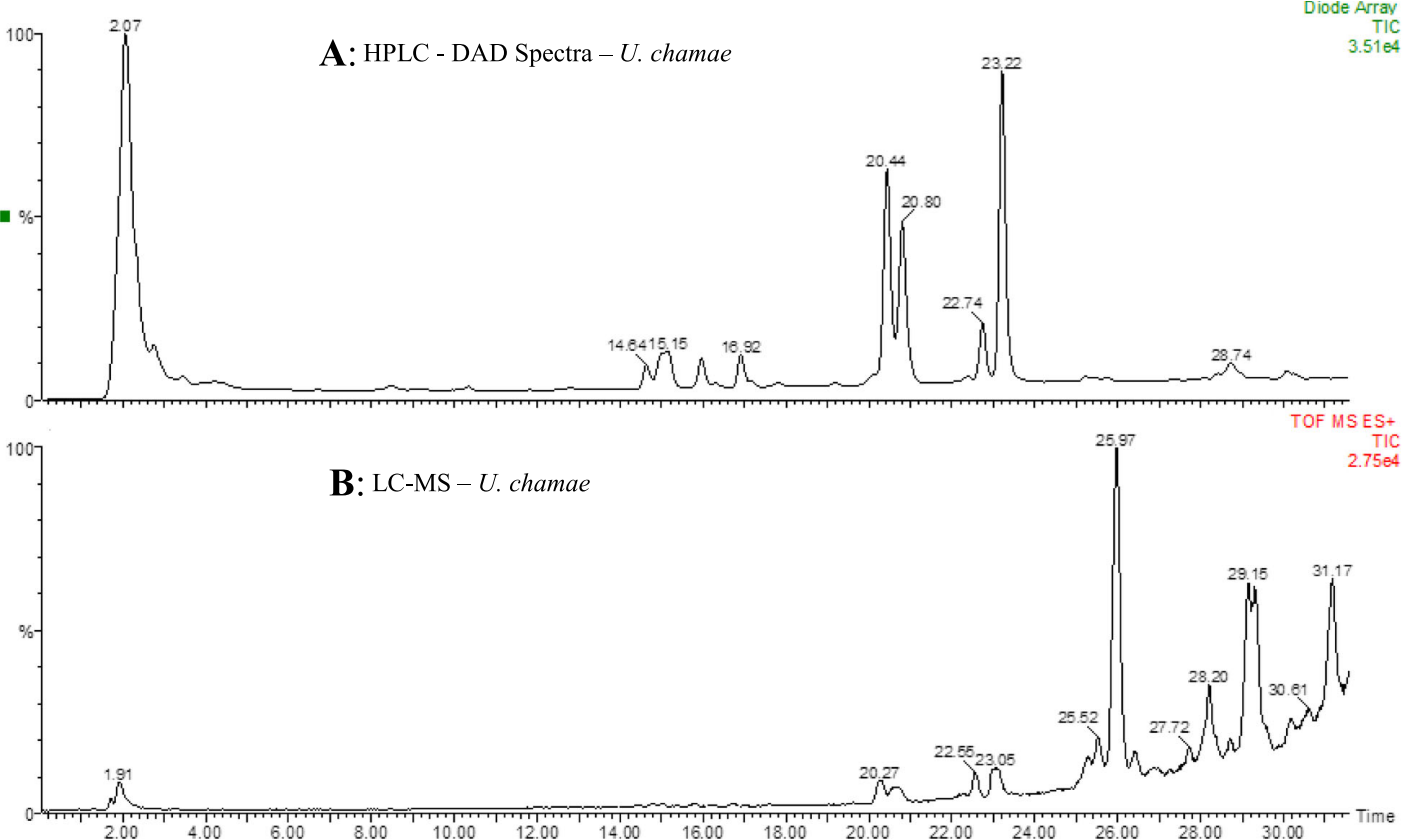
Chromatographic data of *U. chamae* root extract. **A** HPLC-DAD, **B** LC-MS

**Fig. 11:**
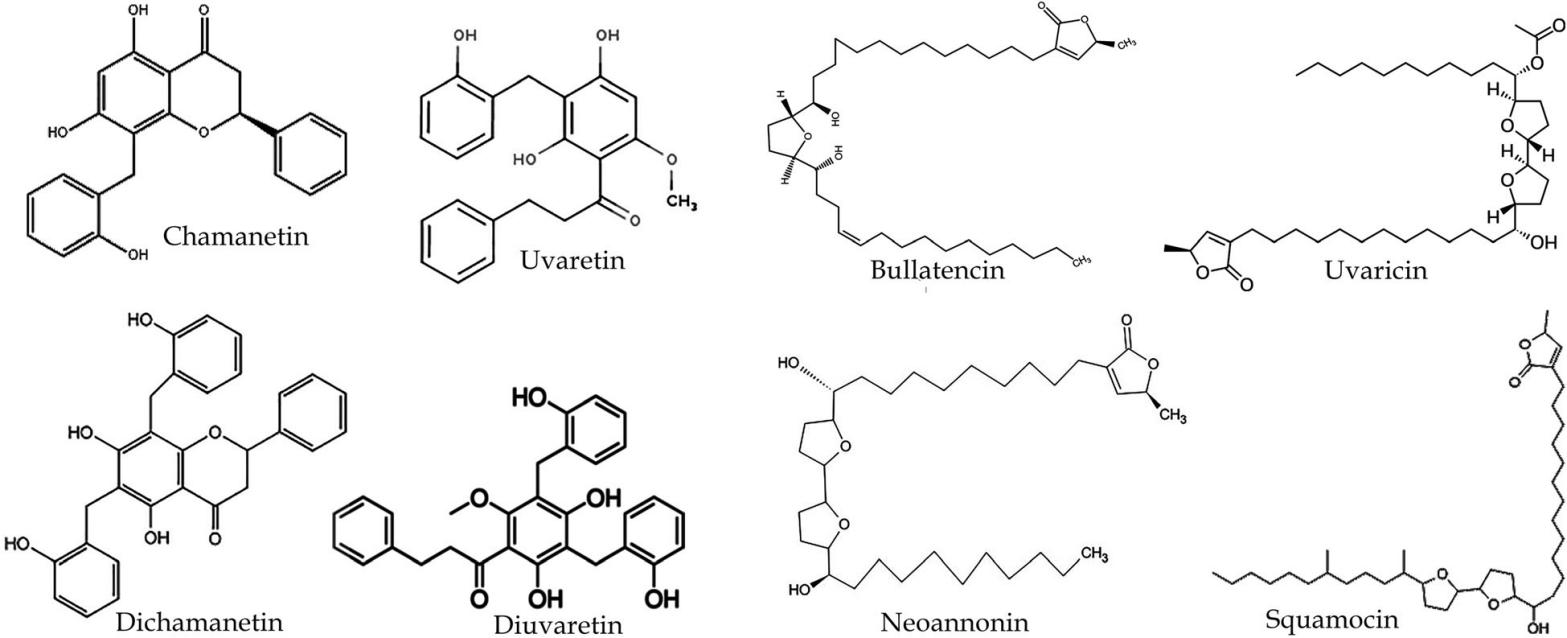
Structure of constituents of *Uvaria chamae* root extract identified by LC-MS

Our approach to medicinal plants research focusing on drug discovery recognises and addresses the need to identify chemical constituents in extracts that might be responsible for their observed biological activities. We, therefore, employed chromatographic and mass spectrometric analyses (coupled) to resolve the constituents of the two extracts investigated. The LC-MS chromatograms for the OS and UC extracts are shown in Figs. 9 and 10, respectively. Analysis of the ion spectra data (m/z for each peak; MS) from the chromatogram of the extract from the root of *U. chamae* revealed the possible presence of bullanin, bullatencin, chamanetin, desacetyluvaricin, dichamanetin, diuvaretin, isochamanetin, isouvaretin (chamuvarin), neoannonin, squamocin, uvaretin, uvaricin I and uvaricin II (Table 2), the structures of some of which are shown in Fig. 11. Ion spectra data from MS chromatogram of *O. subscorpioidea* did not match any previously reported compounds in the plant.

**Table 2:** LC-MS identification of compounds in *U. chamae* roots

	Retention Time (min)	m/z	Possible match^a^	References
1	14.743	363.1907	Chamanetin, Isochamanetin	[20]
2	15.760	385.1786	Chamanetin, Isochamanetin	[20]
3	16.744	401.2128	Isouvaretin/(Chamuvarin) Uvaretin	[21] [22]
4	20.246	401.2128	Isouvaretin (Chamuvarin)	[21]
5	20.630	491.2543	Dichamanetin	[20]
6	22.531	613.3497	Bullatencin	[23]
7	23.031	507.2805	Diuvaretin	[22]
8	25.949	645.5989	Squamocin	[24]
9	27.700	601.5641	Neoannonin	[25]
10	28.701	629.6179	Uvaricin I/II	[26]
11	29.134	629.6061	Desacetyluvaricin	[24]

^a^The compounds were identified by comparing m/z data (ion mass) of the sample with the values reported in the literature

## Discussion

This study provides a key evidence base for the use of two plants, *Uvaria chamae and Olax subscorpioidea*, in the traditional treatment of cancers, and identifies them as potential sources of anti-cancer compounds. The findings are consistent with the exceptional success in identifying anti-cancer principles or their precursors from medicinal plants, as more than 60% of clinically-approved anticancer drugs are derivatives of medicinal plants [27]. The importance, relevance and timeliness of this work could be further appreciated upon consideration that 80% of the world’s population depend on traditional medicines to date [28]. Oxidative stress and inflammation, both of which mechanisms are unequivocally linked to the initiation, promotion and progression of cancers, were the focus of this work, and we demonstrated that the plants were able to combat these patho-mechanisms, although to varying degrees.

Assessment of viability confirmed that the extracts at the concentrations used in the subsequent antioxidant assays (in HeLa and AREc32 cells) and anti-inflammatory assays (in RAW264.7 cells) were generally non-cytotoxic, and it is possible that the profile of mild, non-concentration-dependent toxicity seen at any extract concentration below 100 μg/ml could have been a result of some assay artefacts, a view corroborated by a correlation of the viability data with the accompanying level of morphological damage as shown by the photomicrographs in which extract concentrations up to 100 μg/ml did not reveal any discernible morphological damage, while 200 μg/ml or higher for some extracts demonstrated overt toxicity. We were, therefore, able to establish that lower concentrations of the extracts could be useful as adjunct chemoprevention and/or chemotherapy, while the effects of higher concentrations were consistent with potential use in cytotoxicity-dependent chemotherapy. However, it is important to assess in the future whether the lower concentrations that were not directly cytotoxic in our investigation could induce direct toxicity if the cancer cells were exposed to them for a longer duration.

The extracts demonstrated potent antioxidant effects by directly combatting the levels of ROS induced by hydrogen peroxide and also by activating the cellular Nrf2-ARE antioxidant defence system. Oxidative stress contributes to the initiation, promotion and progression of neoplasms [29]; reducing oxidative stress is thus important, especially in cancer prevention. Over the years, phytochemicals have shown promising chemopreventive effects in several cancer types as a result of their antioxidant properties [30]. Documented in vitro and in vivo data show that, in addition to preventing oxidative damage, antioxidants may alter the intracellular redox state, by which they enhance the effects of cytotoxic therapy. Also, through this mechanism, they could selectively inhibit tumour cell growth [31]. Results from the current study show that the two extracts produced significant reductions in basal intracellular ROS levels in HeLa cells, measured over a 24 h period. Hydrogen peroxide traverses the cell membrane fairly rapidly, and its high levels can cause oxidative damage to cells. Also, hydrogen peroxide releases active oxygen species (highly reactive hydroxyl radical) through the Fenton reaction [32, 33]. The hydroxyl radical reacts with biological molecules such as proteins and DNA and is also known to induce lipid peroxidation through the removal of hydrogen atoms from membrane lipids [34–36]. The extracts showed significant reduction of H_2_O_2_-induced ROS levels measured over a 24 h period following initiation of exposure. This result strongly corroborates our previous study which demonstrated the in vivo antioxidant activities of these extracts, particularly their ability to prevent lipid peroxidation [13], which is a key consequence of oxidative stress.

To better understand the mechanisms underlying the antioxidant actions of the extracts, we explored the potential involvement of the Nrf2-ARE signalling pathway, a major cellular antioxidant defence system [14]. The importance of Nrf2 activators was highlighted by the studies of Lee Wattenberg and Paul Talalay, which demonstrated in mouse models that phenolic antioxidants prevented chemical carcinogenesis through the upregulation of enzymes involved in metabolism [37, 38]. It was later established that this effect was a result of the activation of the NRF2 pathway [18]. Thus, inducers of Nrf2 are considered important cancer chemopreventive agents. Nrf2 is a regulatory transcription factor that induces genes that play critical roles in oxidative and xenobiotic stresses [39]. When cells are exposed to reactive oxygen species (ROS), Nrf2 rapidly accumulates in the nucleus and subsequently heterodimerises with members of the small *Maf* protein family to act upon Antioxidant Response Element (ARE) sequences as a transcriptional activator [40, 41]. The ARE is found in the promoter region of genes encoding detoxification enzymes (e.g., Phase II enzymes) and other cytoprotective proteins [42]. The induction of enzymes, such as glutathione S-transferase, NAD(P)H:quinone oxidoreductase, UDP-glucuronosyltransferase, aldehyde dehydrogenase, γ-glutamate cysteine ligase and glutathione synthetase, helps in the deactivation of oxidative toxicants before they cause damage to critical cellular macromolecules [40].

In this study, we evaluated, using the AREc32 luciferase reporter cell line, the induction/activation of Nrf2-ARE by the study plants. The AREc32 cell line is human MCF-7 breast cancer cell line that was stably transfected with a luciferase reporter gene construct under the control of eight copies of rat *Gsta2* AREs in the promoter region, and it is used to assess whether a molecule could elicit antioxidant activity by inducing Nrf2 activation. While OS induced Nrf2 activity at some concentrations, the potent and concentration-dependent Nrf2-inducing activity of UC was quite remarkable and deserves further investigations into the role UC and its constituents may play in the chemoprevention and treatment of diseases where oxidative stress has been implicated.

However, recent studies have shown that the induction of Nrf-2 could be a double-edged sword, thus warranting caution. For example, evidence indicates that Nrf2 can rewire metabolic programmes to promote cancer cell growth and proliferation as well as promote chemo-resistance and radio-resistance [43, 44]. Lau and colleagues showed a positive correlation between Nrf2 levels and resistance of cancer cell lines to cisplatin, doxorubicin, and etoposide [45]. Wang and colleagues also reported that *tert*-butylhydroquinone (tBHQ) pre-treatment results in activation of Nrf2, which leads to survival of neuroblastoma cells treated with cisplatin, doxorubicin and etoposide [46]. While these data suggest that the role of Nrf-2 in specific cancers would need to be ascertained, the ability of the extracts to induce Nrf-2 activation as we have shown in this study lends credence to their potential, as well as the potential of their constituent compounds, to elicit beneficial effects in the chemoprevention of cancers.

Inflammation and cancer were first linked as far back as a century and a half ago when Virchow highlighted the tendency for cancers to occur at sites of chronic inflammation [47]. Epidemiological investigations have also shown that inflammatory diseases are frequently associated with increased risks of certain cancers [48]. Nitric oxide (NO) and prostaglandins are important for provoking and maintaining an inflammatory condition [49], which is why we assessed the ability of the extracts to lower excessive levels of NO, as an indicator of their anti-inflammatory potential. The RAW264.7 murine macrophage cell line used in our work is commonly used to model macrophage-mediated inflammatory events in vitro [50, 51]. To induce NO production, we challenged the cells with lipopolysaccharide (LPS), as its stimulation has been demonstrated to induce iNOS expression as well as to increase NO production and prostaglandin synthesis in RAW 264.7 cells [51–54]. A similar effect of LPS was seen from our results (Fig. 7), as NO levels (measured as nitrite levels) were increased in RAW 264.7 cells following LPS stimulation. Treatment of the cells with OS and UC, however, significantly decreased NO production at all concentrations tested.

Due to relatively low levels of nitrite obtained following stimulation with LPS alone, and in a bid to ensure the effects we assessed were quite physiologically and pathologically relevant, IFN-γ was used to potentiate the NO-releasing action of LPS. This generated a much higher level of nitrite than was obtained with LPS alone. The two extracts each caused a decrease in the NO production induced by stimulation of RAW 264.7 cells with LPS/IFN-γ. The anti-inflammatory effects were confirmed not to be as a result of the cells dying following the treatments, but are proposed to be through suppression of iNOS mRNA and protein expression in LPS-induced RAW 264.7 cells.

While we identified the antioxidant and anti-inflammatory effects of the extracts without evidence of potent direct toxicity, we recognise the importance of investigating in the future longer-time treatments (e.g., 72 h or 96 h), on the basis that their antioxidant and anti-inflammatory effects in cancer cells would be expected to lead ultimately to the death of those cancer cells. It is also important within this context to test in the future for the ability of the extracts to selectively kill cancer cells at concentrations and exposure durations at which they do not affect the viability of normal cells.

LC-MS analysis of UC extract revealed the possible presence of previously documented compounds. The C-benzylated flavanones chamanetin, isochamanetin and dichamanetin have been shown to be effective adjuvants in antibiotic treatment of infections [55, 56]. The insecticidal, larvicidal, antimalarial and fungicidal efficacies of the compounds from *Uvaria* species (particularly chamanetin and dichamanetin) have also been reported [22, 57, 58]. While santalbic acid has been isolated from *O. subscorpioidea* (OS) [59], we could not relate the m/z data obtained for the OS extract with any documented compounds from this plant species. A detailed study in the future will assess the specific contributions of the constituent compounds to the establishment of the biological effects observed in this study.

## Conclusion

This study has demonstrated the Nrf-2-inducing, antioxidant and anti-inflammatory effects of our study plants in validated cell-based models. As these activities are closely linked to the chemoprevention and chemotherapy of cancers, the work has expanded our understanding of the roles of these plant extracts in traditional remedies that employ them in the therapy of cancers. It has also pointed attention in the direction of these plants for drug discovery purposes.

## Supplementary Information

**Additional file 1: Fig S1**. Comparison of the 24 h and 48 h effects of extracts of *Olax subscorpioidea* (OS) and *Uvaria chamae* (UC) on the viability of HeLa cells (*n* = 3).

## Data Availability

Data sets have not been deposited in any repository but are available from the corresponding author upon request.

## Abbreviations

AB: Alamar blue
ARE: Antioxidant response element
DCFDA: 2′,7′ –dichlorofluorescin diacetate
DMEM: Dulbecco’s Modified Eagle Medium
ECACC: European Collection of Authenticated Cell Cultures
FCS: Foetal Calf Serum
HPLC: High-pressure liquid chromatography
IFNγ: Interferon gamma
LC-MS: Liquid Chromatography-Mass Spectrometry
LPS: Lipopolysaccharide
MeOH: Methanol
NEAA: Non-Essential Amino Acids
NO: Nitric oxide
Nrf2: Nuclear factor erythroid 2-related factor like-2
PBS: Phosphate-buffered saline
ROS: Reactive oxygen species
tBHQ: *Tert*-butylhydroquinone
UV/DAD: Ultraviolet Diode-Array Detector
