# Supplementary material for: Potent Nrf2-inducing, antioxidant, and anti-inflammatory effects and identification of constituents validate the anti-cancer use of Uvaria chamae and Olax subscorpioidea
Source: BMC Complement Med Ther. 2021 Sep 18;21:234. doi: 10.1186/s12906-021-03404-0 (PMC8449903; doi:10.1186/s12906-021-03404-0)
Supplement: Supplementary file 1 — Additional file 1: Fig S1. Comparison of the 24 h and 48 h effects of extracts of Olax subscorpioidea (OS) and Uvaria chamae (UC) on the viability of HeLa cells (n = 3). [file 12906_2021_3404_MOESM1_ESM.pdf]

Comparison of the 24 h and 48 h effects of extracts of *Olax subscorpioidea* (OS) and *Uvaria chamae* (UC) on the viability of HeLa cells (n=3)

**Conc. (µg/ml)**	**OS**		**UC**	
	**24 h**	**48 h**	**24 h**	**48 h**
0	100.00±0.00	100.00±0.00	100.00±0.00	100.00±0.00
5	101.62±4.80	101.58±1.00	111.46±2.77	94.36±2.14
10	99.28±1.71	104.61±2.56	104.24±3.68	98.61±4.27
15	91.19±4.09	98.19±2.67	113.86±5.58	102.12±5.68
25	82.63±5.37	85.15±9.81	104.38±3.91	102.02±2.42
50	75.10±2.68	75.25±12.82	97.57±2.91	101.20±5.71
75	73.16±2.70	71.26±14.22	93.62±3.12	96.73±8.30
100	72.88±2.74	67.73±15.31	84.32±3.77	88.81±11.73
200	75.46±2.68	70.41±14.11	51.76±11.96	42.69±26.22
500	19.58±2.28	20.53±3.69	10.92±0.95	7.68±0.55
